# Motor performance as a predictor of blood levels of ammonia and inflammatory biomarkers in patients with liver cirrhosis

**DOI:** 10.1371/journal.pone.0333029

**Published:** 2025-10-08

**Authors:** Constanza San Martín Valenzuela, Juan José Gallego, Amparo Urios, Patricia Correa-Ghisays, Rafael Tabares-Seisdedos, Carmina Montoliu

**Affiliations:** 1 Unit of Personal Autonomy, Dependency, and Mental Disorders Assessment, Faculty of Medicine, University of Valencia - INCLIVA Biomedical Research Institute, Valencia, Spain; 2 Department of Physiotherapy, Faculty of Physiotherapy, University of Valencia, Valencia, Spain; 3 Centro de Investigación Biomédica en Red de Salud Mental (CIBERSAM), Instituto de Salud Carlos III, Madrid, Spain; 4 Department of Pathology, Faculty of Medicine, University of Valencia, Valencia, Spain; 5 INCLIVA, Biomedical Research Institute, Valencia, Spain; National Research Centre, EGYPT

## Abstract

Liver cirrhosis can present with complications such as hyperammonemia and inflammation, and the consequent appearance of minimal hepatic encephalopathy (MHE), which has been associated with poorer motor performance. Our aim was to analyze whether motor impairment would predict blood levels of ammonia and inflammatory factors in cirrhotic patients, independently of cognitive impairment. Blood was extracted from 67 cirrhotic patients from two hospitals in Valencia (Spain) (41.79% with MHE, diagnosis by Psychometric Hepatic Encephalopathy Score). Blood ammonia and plasma levels of interleukins (IL-6, IL-13, IL-18, IL-21, IL-22, IL-23, TNF-α, TGF-β) and chemokines (CCL20, CX3CL1, CXCL13, CCL2) were measured by micro-diffusion and ELISA, respectively. Gait, balance, hand strength, and manual motor speed were evaluated with biomechanical tools. All measurements were performed at University of Valencia, Spain. Motor outcomes were used as predictors in multiple linear regression analysis to determine their predictive capacity on blood ammonia and inflammatory factors. We found that levels of blood ammonia and TNF-α were significantly predicted by the set of motor parameters with a coefficient of determination (R^2^)>0.50, particularly by balance performance through the movement and velocity of Center of Pressure across the balance test. On the other hand, the models for IL-13, IL-21, CX3CL1, IL-6, CCL2, yielded an R^2^ varying between 0.31–0.39. Lastly, R^2^ was between 0.29 and 0.16 for the cytokines CXCL13, IL-22, CCL20, IL-18, and TGF-β. Beside balance outcomes, gait speed and swing phase, as well as grip and lateral pinch strength, and hand motor speed, were frequent predictors in the models calculated. In conclusion, motor performance can predict blood levels of ammonia and cytokines in patients with liver cirrhosis, independently of the cognitive impairment present. Motor impairment could serve as an early, non-invasive indicator of disease severity, indicating the utility of conducting these tests in daily clinical practice and in follow-up studies.

## Introduction

Liver cirrhosis is widely prevalent worldwide, and is associated with high morbidity and mortality [1]. Cirrhosis is characterized by fibrosis and nodule formation of the liver secondary to chronic injury [2], and it evolves from an asymptomatic phase (compensated cirrhosis) to a symptomatic phase (decompensated cirrhosis), with complications such as ascites, oesophageal variceal bleeding, and hepatic encephalopathy (HE) [1,3]. HE encompasses a broad spectrum of cognitive, psychomotor, and psychiatric disturbances [4], ranging from minimal hepatic encephalopathy (MHE), to grade IV according to symptoms severity by West Haven criteria [3]. Another common system, from the International Society for Hepatic Encephalopathy and Nitrogen Metabolism, divides HE into covert (MHE and grade I) and overt (grades II to IV) forms [5]. Overt HE is present in 10–14% of patients at cirrhosis diagnosis [3] and occurs in 30–40% over the disease course, often recurrently [6]. MHE is the mildest form of HE, and includes a spectrum of brain-related symptoms [7], such as impairment in attention, executive function, visuo-construction, processing speed, motor speed, and motor accuracy [8]. Since these neuropsychiatric features are not exclusive to MHE, diagnosis relies on neurocognitive tests, of which the psychometric hepatic encephalopathy score (PHES) is the gold standard [9] where normative values are available. In our studies, we found that 42% of cirrhotic patients have MHE according to PHES score [10], which is similar to that found in a multicenter study from Europe and the United States [11] reporting a MHE prevalence of 35%, which varied substantially between diseases stages. A study form India showed a MHE prevalence of 59.7% [12] and the prevalence of covert HE in cirrhotic patients in China was 50.4%, gradually increasing with the severity of impaired liver function [13].

Motor impairments have also been reported in patients with HE and MHE, which correlated with their cognitive performance [3,14,15]. Although there are not reported the prevalence of motor impairment in patients with MHE, we found that 80% of MHE patients and 43% of patients without MHE show alterations in motor coordination tests [10]. Moreover, compared to individuals without MHE, MHE patients exhibit poorer gait and balance performance, as well as motor slowing and worse hand strength variability [14]. In addition, both myosteatosis (excessive fat infiltration in the skeletal muscle), and sarcopenia (loss of skeletal muscle mass and strength) seem to be strongly associated with the presence of MHE, as well as with the development of overt HE [16]. Due to this disease pattern, MHE is associated with decreased survival and high risk of recurrence [17], and impacts the lives of both patients and caregivers by worsening daily function, socioeconomic status, and quality of life [18]. Lastly, cognitive decline and psychomotor alterations (e.g., asterixis, dyspraxia) themselves also entail a high risk of progression to overt HE [17].

The major mechanisms implicated in the pathogenesis of HE in cirrhosis include the effects of toxins and pro-inflammatory processes, which are significant for structural and functional brain integrity [4]. Ammonia is one of the main factors related to HE. Due to liver failure, other organs take on roles in the transformation of ammonia into glutamine, such as skeletal muscle, kidneys and brain [19]. In the brain, astrocytes undergo a series of morphological changes compatible with Alzheimer’s disease [20,21], and subsequent elevation of intracellular glutamine. This generates osmotic stress that causes astrocytes to swell [22], dysregulation of metabolic pathways in astrocytes, oxidative stress, and cerebral edema [23].

Besides ammonia, circulating chemokines and cytokines are also increased following liver injury, leading to activation of microglia and a subsequent neuroinflammatory response [23]. The systemic release of pro-inflammatory cytokines and mediators including but not limited to Tumour Necrosis Factor alpha (TNF-α) and interleukins (IL) such as IL-1β, IL-6, IL-8 and IL-12, may aggravate the condition of patients with cirrhosis and precipitate or exacerbate HE [19]. Therefore, peripheral inflammation and hyperammonemia play synergistic roles in inducing MHE in cirrhotic patients [24,25], indicating that inflammation exacerbates the neuropsychological alterations induced by hyperammonemia [26]. The prevalence of high ammonia and ILs levels in MHE patients is not reported, but some studies showed that patients with MHE have elevated levels of proinflammatory interleukins in plasma compared to patients without MHE [24,25,27,28]. Moreover, MHE appearance is associated with specific changes in the immune system and peripheral inflammation [29].

Studies have demonstrated that arterial and venous ammonia is significantly correlated with HE severity, as measured by the West Haven Criteria [30], yet no significant relationship between the PHES score and blood ammonia has been found [31]. In this way, Giménez et al. reported that PHES is not sensitive enough to detect early neurological alterations in a significant proportion of cirrhotic patients [10]. Due to the previously demonstrated association of motor decline with onset of MHE [14], in this work we aimed to analyze whether motor performance in cirrhotic patients with and without MHE could be a statistical predictor of plasma biomarker levels such as ammonia and inflammatory factors. Our hypothesis was that motor impairment is progressive and is related to toxicity and inflammation in patients with liver cirrhosis, regardless of whether they have cognitive impairment diagnosed via PHES. If so, motor impairment may represent a new area indicating the ammonia and inflammatory stage of patients with liver cirrhosis. Given that this motor impairment is easily observable and reportable in daily clinical practice without the need for test batteries or laboratory analysis, this could potentially enhance early diagnosis and treatment of MHE, by helping clinicians prioritize specific diagnostic tests for patients with motor impairment.

## Materials and methods

### Study design, setting, and participants

This study is cross-sectional with a convenience sampling approach (specifically, modal instance sampling, i.e., volunteers with the pathology for study). It was designed following STROBE guidelines (S1 Table). A total of 67 participants with liver cirrhosis were consecutively recruited from 4 March 2018–12 November 2024, at the outpatient clinics of Hospital Clínico and Hospital Arnau de Vilanova in Valencia, Spain. Diagnosis of cirrhosis was based on clinical, biochemical and ultrasonographic data. Inclusion criteria were (i) compensated liver cirrhosis, (ii) able to stand and walk without use of assistive devices, (iii) stable medication and (iv) over 18 years old. Exclusion criteria were (i) overt HE or history of overt HE, (ii) recent (<6 months) alcohol intake, (iii) history of abuse or drug-dependence of any another substances besides alcohol, (iv) history of disease or secondary trauma that could influence cognitive or motor deterioration, (v) chronic disease without normative treatment (visual problems, high blood pressure, diabetes, hypercholesterolemia), (vi) established neurological or psychiatric disorders. All participants were included after giving written informed consent. Study protocols were approved by the Scientific and Research Ethics Committees of Hospital Clínico Universitario and Arnau de Vilanova Hospital of Valencia, Spain (approval code: 2018.051; approval date: 27 February 2018; approval code: 2023/130; approval date: 29 February 2024) and were in accordance with the principles of the World Medical Association’s Declaration of Helsinki, the Council of Europe Convention regarding human rights and the requirements established in Spanish legislation in the field of Biomedical research, personal data protection and bioethics.

### Variables and measurement

Recruited study participants who had signed informed consent underwent blood extraction after fasting. On the same day, a clinical interview was performed to record participants’ baseline characteristics, such as weight, height, and cognitive status. Thereafter, venous blood ammonia level was measured immediately after blood extraction with the Ammonia Test Kit II for the PocketChem BA system (Arkray, Inc., Kyoto, Japan) following the manufacturer’s instructions. Blood samples were centrifuged for 10 min at 1500 g, and plasma was kept at −80 °C for subsequent cytokine analysis. Concentrations of cytokines were measured by ELISA (R&D Systems, Minneapolis, MN, USA) according to the manufacturer’s instructions. Beside ammonia (μM), the inflammatory parameters (pg/mL) studied were TNF-α, interleukins IL-6, IL-13, IL-18, IL-21, and IL-22; fractalkine (CX3CL1); chemokines CCL2 and CCL20; chemokine ligand 13 (CXCL13), and transforming growth factor beta (TGF-β). The relevance of these parameters in liver cirrhosis has been previously reported [27,29]. Blood analytical parameters, including glucose levels, electrolytes, and hepatic enzymes, were collected to characterize the participants.

Additionally, cognitive status was also assessed on the same day as the blood collection in order to characterize the sample. All participants completed the Psychometric Hepatic Encephalopathy Score (PHES) [9,32,33], which was calculated, adjusting for age and educational level, using Spanish normality tables (www.redeh.org/TEST_phes.htm, accessed on 19 July 2023). Patients were classified as MHE when the score was ≤−4 [32]. Severity of liver disease indicators was calculated using the Child-Pugh score and the model end-stage liver disease (MELD) score after etiology data related to the liver disease and analytical values were collected.

All patients performed the motor tests in the morning, at the same time (9:00 am) and under the same conditions (non-fasting). Given the time required to carry out motor tests, motor performance was assessed in a different session, but no later than one week after blood extraction, depending on the availability of the participant, in order to avoid possible progression of the pathology and distortion of the results. Gait, balance, hand strength, and manual motor speed were evaluated with instrumental and biomechanical tools. Motor outcomes (shown in Table 1) were used as predictor variables in the analyses. First, gait was measured using two photocells and two force platforms (Dinascan/IBV Biomechanical Institute of Valencia, Valencia, Spain; and NedAMH/IBV software version 5.1.0, 2013, Biomechanical Institute of Valencia, Valencia, Spain). Participants walked barefoot along a 10 m-long corridor at a self-selected comfortable speed. The platforms were located at the center of the corridor to record the central step and avoid acceleration and deceleration at the start and end of the gait cycle. Secondly, functional postural balance was assessed using a single force platform and NedSVE®/IBV software (version 5.1.0, 2013, Biomechanical Institute of Valencia, Valencia, Spain) [34]. Center of Pressure (CoP) displacement was recorded during Romberg tests for 30 s under four different stability conditions (eyes open or closed, standing on a firm surface or on a 10 cm-thick foam pad) [35]. During the tests, participants maintained a standing position and marks were used on the pad to standardize foot position [14].

**Table 1:** Motor outcome parameters measured used as predictors in the study.

*Gait assessment*	
Gait velocity (m/s)	Distance traveled during a period of time
Stance time (s)	Period of time when the foot is in contact with the ground
Braking force (N)	Minimum registered anteroposterior GRF corresponding to heel strike
Propulsive force (N)	Maximum registered anteroposterior GRF corresponding to take-off
Swing force (N)	Minimum registered vertical GRF occurring during contralateral foot oscillation
Push-off force (N)	Second highest registered of vertical component of GRF between heel-off and toe-off
*Static balance assessment*	
CoP velocity (m/s)	Mean velocity of CoP projection from start to end of test (total distance traveled by the CoP/ time elapsed)
CoP displacement angle (º)	Displacement vector orientation extending from the starting point to the end position of the subject
CoP displacement (mm)	Total distance of the CoP reached from the origin. Maximum anteroposterior and mediolateral distance reached by the CoP
CoP dispersion (mm)	Dispersion of the point cloud described by the CoP in the mediolateral and anteroposterior directions
CoP force (N)	Maximum force in mediolateral and anteroposterior directions exerted by the CoP during the test
CoP swept area (mm^2^)	Area of subject’s swing, calculated from beginning until end of the test
*Manual motor speed assessment*	
FTT right and left (freq.)	Number of times the key is pressed with the right and left hand
FTT2 right and left (freq.)	Number of times the key is pressed with the right and left hand while the test is performed with both hands
*Hand strength assessment*	
Grip strength (N)	Average of grip strength repetitions, for right and left hand individually
Lateral pinch strength (N)	Average of lateral pinch strength repetitions, for right and left hand individually
Tip pinch strength (N)	Average of tip pinch strength repetitions, for right and left hand individually
Coefficient of variation (%)	Standard deviation ratio to mean hand strength during each task, which indicates variability in hand force strength performance and strength stability control
Index of Difference (ID)	Difference in performance between right and left hand

Motor outcomes registered per test: m/s, meter/second; s, second; N, newton; GRF, ground reaction force; CoP, center of pressure; mm, millimeters; mm^2^, square millimeters; FTT, finger tapping test; freq., frequency.

After gait and balance, participants performed hand motor tests sitting upright in a chair with a backrest but without armrests. Feet were supported on the floor with 90º knee flexion, and arms positioned with 90º elbow flexion. First, motor speed was assessed through the Finger Tapping Test (FTT). Each subject was required to tap a key as many times as possible using the index finger of each hand, and the number of taps within 30 s was recorded [36]. Participants completed the test once with the right hand, again with the left hand and lastly performed the test with both hands at the same time. Finally, grip strength was measured with an electronic dynamometer (NedVEP/IBV Biomechanical Institute of Valencia, Valencia, Spain) and NedDiscapacidad/IBV software (version 4.1.1, Biomechanical Institute of Valencia, Valencia, Spain) [37,38]. The forearm was placed in neutral pronosupination position [39] to perform three functional force gestures for 30 s: a) handgrip, b) lateral pinch (thumb pad and side of index finger), and c) tip pinch (thumb and index finger) [14]. For each gesture, three repetitions of maximum force were obtained, ensuring they did not differ by more than 10% [40].

### Bias and study size

The main bias detected in the study was the presence of comorbidities adding an inflammatory burden beyond that generated exclusively by the disease. This bias was controlled through the participation criteria, the clinical interview conducted and participants’ medical history. Sample size estimation was performed for the statistical analysis presented in this manuscript. For this purpose, the G*Power software (Universität Kiel, Germany, Version 3.1) [41] was used, applying and alpha level of 0.05 and a power of 0.8. The expected effect size was set at R² = 0.5, indicating that at least 50% of the variance in the biological variables would be explained by the 19 motor predictors included in the analysis. Based on this calculation, a total of 58 participants were required.

### Statistical analysis

Statistical analyses were performed using IBM SPSS V26 (SPSS Inc., Chicago, IL, USA). All motor outcomes served as the independent variables in this study and were used as predictors in the analyses discussed below. In contrast, the registered biochemical variables (from blood test) constituted the dependent variables, that is, the predicted outcomes for which a separate regression model was generated. Descriptive data (mean, standard deviation, and range) were used to inform the continuous outcomes with normal distribution. Variables not meeting this assumption were reported using median and range. Categorical variables were reported through frequencies. As the assumption of normality was met in the variables analyzed and residuals, a stepwise multiple linear regression analysis model was used for the ammonia and biochemical markers measured. Given the large number of predictors relative to the number of participants, stepwise regression was employed to obtain a regression model with the minimum number of statistically significant predictors [42]. The multiple correlation coefficient squared value (R^2^) was calculated for each regression model to demonstrate goodness of fit. Beta coefficient values (β), standard error (SE), and significance values for each regression model were also reported.

## Results

The assessments of the study were completed for 67 participants, whose characteristics, etiology of cirrhosis and analytical parameters are shown in Table 2. Across the whole sample, 28 patients (41.79%) were classified as having MHE versus 39 patients (58.21%) without MHE, according to PHES score. Blood ammonia and plasma levels of interleukins (IL-6, IL-13, IL-18, IL-21, IL-22, IL-23, TNF-α, TGF-β) and chemokines (CCL20, CX3CL1, CXCL13, CCL2) tested are shown in S2 Table, as well as their comparison with reference data. Cirrhotic patients showed significantly higher blood ammonia levels (p < 0.05) and altered plasma levels of ILs measured, in contrast with non-diseased blood levels data.

**Table 2:** Clinical and demographic characteristics of the participants.

Demographical and Clinical variables	Cirrhotic patients (n = 67)	
Sex (Male/Female)^a^	52 (77.61%)/15 (22.39%)	
Age (years)^b^	62.31 ± 8.82 (46-86)	
Weight (kg)^b^	80.35 ± 13.85 (54.5-113.1)	
Height (m)^b^	166.56 ± 7.75 (148-184)	
BMI^b^	29.01 ± 4.55 (19.50-38.79)	
Child-Pugh score (A/B/C)^a^	49/16/2	
MELD score^b^	9.49 ± 2.78 (5-17)	
Age at diagnosis of cirrhosis^c^	55 (49-63)	
Time from diagnosis of cirrhosis to inclusion in the study (years)	3 (1-7.5)	
Etiology of cirrhosis^a^		
Alcohol	31	
HBV/HCV/HCV + alcohol	1/17/3	
MASH	12	
Other	3	
Analytical parameters^b^		Normal range
Glucose (mg/dL)	114 ± 39	64-106
Sodium (mmol/L)	139 ± 3	135-145
Potassium (mmol/L)	4.3 ± 0.6	3.5-5.1
AST (U/L)	41 ± 21	11-34
ALT (U/L)	31.6 ± 13.5	<34
GGT (U/L)	92 ± 78	9-36
PHES^b^	−3.98 ± 4.50 (−14−3)	
MHE/ No MHE^a^	28/ 39	

^a^Values are expressed as frequency. ^b^Values are expressed as mean ± standard deviation (range).^c^data expressed as median (Interquartile range). AST, aspartate transaminase; ALT, alanine transaminase; BMI, body mass index; HBV, hepatitis B virus; HCV, hepatitis C virus; GGT, γ-glutamyl transpeptidase; MASH, metabolic dysfunction-associated steatohepatitis; MELD: model end-stage liver disease. PHES: Psychometric Hepatic Encephalopathy Score. MHE, no MHE: patients with and without minimal hepatic encephalopathy, respectively, according to PHES score.

We previously evaluated whether some potential confounders such as blood electrolytes and the presence of diabetes, produced differences in motor performance and in the values of the blood parameters measured. As shown in Table 2, all patients in this study showed normal levels of sodium and potassium, and these variables would not influence motor function in the patients in our study.

We also analyzed whether the presence of diabetes could influence differences in motor performance and in the values of the blood parameters measured. The presence of diabetes did not lead to statistically significant differences in either motor performance or the blood biomarkers analyzed (S3 and S4 Tables). Therefore, we consider that the presence of diabetes is unlikely to have influenced our results.

Other possible confounder could be the alcoholic etiology, however we previously showed that there were no significant differences in parameters of motor performance measured between cirrhotic patients of alcoholic etiology and other etiologies [14].

The model that explained the greatest variance in each biomarker was selected from stepwise analysis. For IL-23, no statistically significant model was found (p > 0.05). For the biomarkers with a significant result (p < 0.05), the regression models calculated explained between 16 and 59% of the biomarker data dispersion. Table 3 shows the results for each model. Ammonia (F_(8.56)_ = 10.20, p < 0.01) and TNF-α (F_(7.57)_ = 8.25, p < 0.01) outcomes achieved models with an R^2^ > 0.50 (Table 3). In the ammonia model, variables related to balance and gait functioned as predictors, while in the TNF-α model gait, balance and hand strength variables were significant outcome predictors. From the ammonia model, the most meaningful outcomes were CoP force (SBeta = 0.75, p < 0.01) and dispersion (SBeta = 0.91, p < 0.01) in the anteroposterior direction during the Romberg test with eyes open (Table 4). According to the unstandardized coefficients reported in Table 4, a one-unit increase in CoP dispersion in the anteroposterior direction during the Romberg test with eyes open is associated with an average increase of 13.54 units in ammonia concentration, holding all other variables from the model constant. Likewise, a one-unit increase in CoP force in the anteroposterior direction during the same Romberg test is associated with an average increase of 8.14 units in ammonia concentration.

**Table 3:** Summary of multiple linear regression models for each biomarker measured.

Biomarker	R	R^2^	F	*p*	β_0_
Ammonia	0.77	0.59	10.20	0.00	105.51
TNF-α	0.71	0.50	8.25	0.00	4.98
IL-13	0.63	0.39	7.62	0.00	8.65
IL-21	0.61	0.38	9.08	0.00	1078.15
CX3CL1	0.60	0.36	11.62	0.00	4011.32
IL-6	0.56	0.32	14.64	0.00	10.45
CCL2	0.56	0.31	6.80	0.00	41.84
CXCL13	0.54	0.29	8.35	0.00	209.29
IL-22	0.53	0.28	11.87	0.00	67.92
CCL20	0.51	0.26	7.09	0.00	87.73
IL-18	0.41	0.18	6.56	0.00	1423.93
TGF-β	0.40	0.16	5.97	0.00	8756.13

The regression models for each biomarker are shown in order of explained variance. The multiple correlation coefficient (R) and squared value (R^2^) are indicated. The Fisher F ratio and its significance value (*p*) from the ANOVA analysis, as well as the β_0_ coefficient are also reported.

**Table 4:** Outcome predictors from the multiple regression models that explain over 50% of the variance in the biomarkers analyzed.

Biomarker	Predictor	Unstandardized		Stand.	CI	t	*p*
		β	SE				
AMMONIA	AP CoP Force in REO	8.14	1.39	0.75	5.36; 10.93	5.86	0.00
	AP CoP Dispersion in REO	13.54	2.47	0.91	8.58; 18.49	5.47	0.00
	Displacement CoP angle in RFC	0.06	0.03	0.21	0.00; 0.13	2.10	0.04
	AP CoP Force in RFC	0.54	0.16	0.30	0.22; 0.86	3.38	0.00
	AP CoP Force in RFO	1.57	0.42	0.35	0.71; 2.42	3.67	0.00
	Swing GRF during gait	0.09	0.03	0.36	0.15; 0.03	−3.00	0.00
	Propulsive GRF during gait	−0.31	0.11	−0.30	−0.53; −0.09	−2.83	0.00
	ML CoP Force in REO	4.27	2.03	0.44	0.19; 8.34	2.09	0.04
TNF-α	Push-off GRF during gait	−0.02	0.01	−0.19	−0.00; 0.00	−1.96	0.05
	CoP Velocity in RGC	17.03	4.85	0.34	7.31; 26.75	3.50	0.00
	Total CoP displacement in REC	0.08	0.03	0.34	0.03; 0.13	3.53	0.00
	Total CoP displacement in RFC	0.45	0.02	0.27	0.01; 0.07	2.85	0.00
	Tip pinch strength (left hand)	−0.03	0.01	−0.35	−0.05; −0.01	−3.46	0.00
	Lateral pinch difference index	−0.02	0.00	−0.27	−0.03; −0.01	−2.78	0.00
	Lateral pinch (left) variation coeff.	−0.07	0.03	−0.24	−0.13; −0.02	−2.56	0.01

The standardized and unstandardized β coefficients, their standard error (SE), t values, significance, and confidence interval (CI) are reported for each predictor. CoP, center of pressure; AP, anteroposterior direction; ML, mediolateral direction; GRF, ground reaction force; REC, Romberg test with eyes closed; REO, Romberg test with eyes open; REC, Romberg test with eyes closed; RFO, Romberg with foam pad and eyes open; RFC, Romberg test with foam pad and eyes closed.

In the TNF-α model, while multiple predictors were statistically significant (p < 0.05), CoP velocity during the Romberg test with foam and eyes closed was the only variable with an unstandardized coefficient of meaningful magnitude (SBeta = 0.34, p < 0.01). Accordingly, a one-unit increase in CoP velocity during the aforementioned balance test is associated with a mean increase of 17.03 units in ammonia concentration.

For biomarkers whose models explained less than 50% of variance, the full list of predictors in provided in S5 Table. The most consistently identified predictor across within these models was gait speed, which played a significant role in the models for IL-21 (SBeta = −0.40, p < 0.01), IL-6 (SBeta = −0.50, p < 0.01), CCL20 (SBeta = −0.24, p < 0.05), CXCL13 (SBeta = −0.33, p < 0.01), and IL-18 (SBeta = −0.44, p < 0.01). A negative coefficient for walking speed was observed in all models mentioned, indicating that lower gait speed was consistently linked with higher levels of the interleukins and cytokines analyzed.

Balance performance was also a significant predictor in all the models tested with an R^2^ below 0.50, but Romberg tests and balance outcomes varied between them. From this data set, the most consistently identified predictive outcomes were derived from the Romberg test with eyes closed and subjects standing on a foam pad. These outcomes were included in the models for IL-21, CX3CL1, IL-6, CCL2, CXCL13, and CCL20. The most frequently recurring predictor was the CoP velocity displacement during the test, which showed a positive association with IL-6 (SBeta = .26, p < .01), CCL2 (SBeta = .31, p < .01), and CCL20 (SBeta = .26, p < .05) levels. An increase in CoP velocity was associated with elevated concentrations of these biomarkers. Finally, hand motor performance was statistically predictive variable in the IL-21, CX3CL1, CCL2 and TGF-β models (p < .05). As shown in S5 Table, while the coefficient of variation and the hand force difference index were positively associated with increased levels of IL-21 (SBeta.25, p = .01), CCL2 (SBeta = .29, p = 0.01), and TGF-β (SBeta = .30, p = 0.01), both hand strength and manual speed exhibited a negative relationship with the measured blood parameters.

## Discussion

This study aimed to analyze whether motor performance in patients with liver cirrhosis (with and without MHE) could predict levels of biomarkers such as ammonia and inflammatory factors. Although previous studies have investigated the association of motor outcomes with MHE [14], to our best knowledge, this is the first work to predict blood parameter levels based solely on motor performance in cirrhosis patients with and without cognitive impairment. We found that ammonia and TNF-α levels were predicted with a coefficient of determination higher than 0.50 by the set of motor predictors. First, we observed that blood ammonia and TNF-α were predicted mainly by balance performance, by both movement and velocity of the CoP across the Romberg tests explored. CoP velocity displacement also had a predictive role in IL-6, CCL2 and CCL20 cytokines, although with a lower percentage of variance. Several structures across the brain intervene in postural balance control; however, the cerebellum has a key role in balance acquisition and ability [43]. Our results could indicate that the accumulation of ammonia and inflammatory factors may affect specific brain locations responsible for balance, a theory which has not been previously described. The results of previous research could support this hypothesis. A previous study reported that ammonia and proinflammatory cytokine levels correlated with alterations in posturographic parameters such as RFO and limits of stability in MHE patients [34], highlighting the relationship between hyperammonaemia and inflammation and impaired postural control in patients with MHE. Studies in animal model for MHE showed that plasma extracellular vesicles from hyperammonemic rats were enriched in TNF-α and induced neuroinflammation in cerebellum, as well as motor incoordination in control rats. These effects were reversed by blocking the action of TNF-α [44,45]. Moreover, Felipo et al. reported significantly increased cerebral blood flow in the cerebellar vermis in patients with MHE compared with non-MHE patients, and also observed increased blood flow in cerebellar hemispheres in cirrhotic patients, irrespective of the presence of MHE [46], pointing to the cerebellum as a sensitive region in cirrhosis. Similarly, neuronal cell loss from cerebellum, among other areas, has been reported in hepatocerebral degeneration [4]. Nonetheless, that balance impairment is a predictor in all of the biomarkers tested could also be explained by deterioration in balance-related systems of non-cerebellar causes. One such example is sarcopenia, a common cirrhotic complication which could hamper the inability to respond successfully to challenging postures [47].

Regarding gait speed, this aspect was a predictor for IL-21, IL-6, CCL20, CXCL13 and IL-18, but not for ammonia or TNF-α. In the latter two biomarkers, predictive outcomes from gait were related to the swing phase of the gait cycle and the forces that propel the foot just before the swing. Walking speed has become an indicator of health in many pathologies and is currently widely considered a sixth vital sign [48,49]. The beta coefficients reported in the results section indicate a negative association in which the slower the measured walking speed, the higher the estimated inflammatory factor. Several authors have previously referenced walking speed as an independent risk factor for mortality in people with cirrhosis [50,51], so it was among the variables we expected to be predictive. However, the gait predictors in ammonia and TNF-α showed that a worse stance phase is associated with high values of these biomarkers in blood. This characteristic is extremely important in patients with physical impairment because worse gait quality is directly linked to a higher risk of falls [52], which becomes especially problematic in people with impaired balance, and is reported in patients with MHE [53]. Furthermore, reduced leg swing in gait is related to worse kinematic performance, which in turn may be conditioned by both muscle strength deficiency and neural impairment in gait pattern. Although not evaluated in this study, the sarcopenia characteristic in cirrhotic patients could potentially impact on the importance of this gait milestone in the regression analysis.

Lastly, we observed that strength and speed, along with their difference index and coefficient of variation, play a relevant role as predictors in the models for IL-21, CCL2, and TGF-β. Although handgrip strength has been shown to be decreased in cirrhotic patients in previous studies [54,55], other motor performance outcomes such as hand speed have not been previously informed. In line with our findings, Lai et al*.* [56] developed a liver-specific frailty index in patients with liver disease, which include three performance-based tests of physical frailty: grip strength, chair stands, and balance testing. This index was especially true for those who were older, obese, or had HE or medical co-morbidities [56]. As shown in our results, the hand-related predictors were included in the models with the lowest explained variance, but they still played a relevant role alongside gait speed and balance parameters, which supports this frailty profile as a good reflection of participants’ physiological reserve.

In terms of clinical application, motor assessment should be included in cirrhotic patient exams even prior to cognitive manifestations. Our results suggest that balance assessment should examine both the amplitude of CoP movement and the speed of displacement, an important factor in falls due to balance impairment. Previous studies have reported that falls are prevalent in people with cirrhosis, and commonly lead to loss of independence, reduced quality of life, and mortality [47]. The physical exam should also include gait speed, swing phase of gait cycle and hand grip strength. These physical tests have the advantage of being noninvasive, low cost, and with immediate results that can alert medical professionals to worsening blood biomarkers. Our data support the evidence that impairment of motor variables may represent an early marker of dysfunction in patients with liver cirrhosis, even prior to detectable neuropsychometric changes [14,46]. Follow-up studies should determine the timing of systemic signs and symptoms (both motor and cognitive) occurring in people with cirrhosis as liver disease worsens.

The limitations of our work are that the regression models proposed in this manuscript were made with a local sample, so their external validity must be verified in samples with other sociodemographic characteristics. Likewise, the study lacks a secondary sample percentage to test the regression models generated in the initial sample. This omission prevents the completion of a cross-validation procedure, which is necessary to determine whether the original models can be generalized to other samples. Future studies should incorporate this procedure if they intend to analyze how motor performance are related to inflammation in people with liver cirrhosis.

## Conclusions

The blood levels of ammonia and cytokines in patients with liver cirrhosis can be predicted through clinical motor deterioration, independently of patients’ cognitive impairment. Blood ammonia and TNF-α levels can be predicted by motor variables with more than 50% of variance explained. Movement and velocity of CoP during balance tests, gait speed and swing phase, as well as grip and lateral pinch strength, in addition to hand motor speed, were repeated predictors in the models calculated for the different parameters analyzed in blood. Motor impairment may represent an alternative area to provide useful information on the pathophysiological state associated with liver cirrhosis, thus warranting evaluation in daily clinical practice and future follow-up studies.

## Supporting information

S1 Table STROBE Checklist. Items reported in cross-sectional study manuscripts, with corresponding page and line references where each item is covered.
(DOCX)

S2 Table Blood ammonia and plasma levels of interleukins and chemokines tested in the sample of participants and a control group as a reference. (DOCX)

S3 Table Differences between sample participants with and without diabetes on parameters from blood test. (DOCX)

S4 Table Differences between sample participants with and without diabetes on motor outcomes. (DOCX)

S5 Table Outcome predictors from the multiple regression models that explain less 50% of the variance in the biomarkers analyzed. (DOCX)
