# Supplementary material for: Motor performance as a predictor of blood levels of ammonia and inflammatory biomarkers in patients with liver cirrhosis
Source: PLoS One. 2025 Oct 8;20(10):e0333029. doi: 10.1371/journal.pone.0333029 (PMC12507304; doi:10.1371/journal.pone.0333029)
Supplement: S1 Table — Items reported in cross-sectional study manuscripts, with corresponding page and line references where each item is covered. (DOCX) [file pone.0333029.s001.docx]

**Motor performance as a predictor of blood levels of ammonia and inflammatory biomarkers in patients with liver cirrhosis**

Constanza San Martín Valenzuela^¶^, Juan José Gallego^¶^, Amparo Urios, Patricia Correa-Ghisays, Rafael Tabares-Seisdedos^*^, Carmina Montoliu^*^

**S1 Table: STROBE Checklist. Items reported in cross-sectional study manuscripts, with corresponding page and line references where each item is covered**

|  | **Item No** | **Recommendation** | **Line / page** |
| --- | --- | --- | --- |
| **Title and abstract** | 1 | (*a*) Indicate the study’s design with a commonly used term in the title or the abstract | 1-2 / 1 |
|  |  | (*b*) Provide in the abstract an informative and balanced summary of what was done and what was found | 21-45 / 2 |
| **Introduction** | | |  |
| Background/rationale | 2 | Explain the scientific background and rationale for the investigation being reported | 49-123/ 3-6 |
| Objectives | 3 | State specific objectives, including any prespecified hypotheses | 112-118 / 5-6 |
| **Methods** | | |  |
| Study design | 4 | Present key elements of study design early in the paper | 127-129 / 6 |
| Setting | 5 | Describe the setting, locations, and relevant dates, including periods of recruitment, exposure, follow-up, and data collection | 129-131 / 6 |
| Participants | 6 | (*a*) Give the eligibility criteria, and the sources and methods of selection of participants | 129-140/6 |
| Variables | 7 | Clearly define all outcomes, exposures, predictors, potential confounders, and effect modifiers. Give diagnostic criteria, if applicable | 134-210/7-10  Table 1 |
| Data sources/ measurement | 8* | For each variable of interest, give sources of data and details of methods of assessment (measurement). Describe comparability of assessment methods if there is more than one group | 134-210/7-10  223-238 / 10-11 |
| Bias | 9 | Describe any efforts to address potential sources of bias | 212-215 / 10 |
| Study size | 10 | Explain how the study size was arrived at | 105-221/10 |
| Quantitative variables | 11 | Explain how quantitative variables were handled in the analyses. If applicable, describe which groupings were chosen and why | 134-210/7-10 |
| Statistical methods | 12 | (*a*) Describe all statistical methods, including those used to control for confounding | 223-238 / 10-11 |
|  |  | (*b*) Describe any methods used to examine subgroups and interactions |  |
|  |  | (*c*) Explain how missing data were addressed |  |
|  |  | (*d*) If applicable, describe analytical methods taking account of sampling strategy |  |
|  |  | (*e*) Describe any sensitivity analyses |  |
| **Results** | | |  |
| Participants | 13* | (a) Report numbers of individuals at each stage of study—eg numbers potentially eligible, examined for eligibility, confirmed eligible, included in the study, completing follow-up, and analysed | 119-129 / 6 |
|  |  | (b) Give reasons for non-participation at each stage | N/A |
|  |  | (c) Consider use of a flow diagram |  |
| Descriptive data | 14* | (a) Give characteristics of study participants (eg demographic, clinical, social) and information on exposures and potential confounders | 129-140 / 6 Table 2 |
|  |  | (b) Indicate number of participants with missing data for each variable of interest | N/A |
| Outcome data | 15* | Report numbers of outcome events or summary measures | 241-322 / 11-16  Table 3; Table 4 |
| Main results | 16 | (*a*) Give unadjusted estimates and, if applicable, confounder-adjusted estimates and their precision (eg, 95% confidence interval). Make clear which confounders were adjusted for and why they were included | 241-322 / 11-16 |
|  |  | (*b*) Report category boundaries when continuous variables were categorized | - |
|  |  | (*c*) If relevant, consider translating estimates of relative risk into absolute risk for a meaningful time period | - |
| Other analyses | 17 | Report other analyses done—eg analyses of subgroups and interactions, and sensitivity analyses | - |
| **Discussion** | | |  |
| Key results | 18 | Summarise key results with reference to study objectives | 324-386/ 16-18 |
| Limitations | 19 | Discuss limitations of the study, taking into account sources of potential bias or imprecision. Discuss both direction and magnitude of any potential bias | 400-407/ 19 |
| Interpretation | 20 | Give a cautious overall interpretation of results considering objectives, limitations, multiplicity of analyses, results from similar studies, and other relevant evidence | 324-386/ 16-18 |
| Generalisability | 21 | Discuss the generalisability (external validity) of the study results | 387-399/ 18-19  409-417/19 |
| **Other information** | | |  |
| Funding | 22 | Give the source of funding and the role of the funders for the present study and, if applicable, for the original study on which the present article is based | Funding online |
