# Supplementary material for: Motor performance as a predictor of blood levels of ammonia and inflammatory biomarkers in patients with liver cirrhosis
Source: PLoS One. 2025 Oct 8;20(10):e0333029. doi: 10.1371/journal.pone.0333029 (PMC12507304; doi:10.1371/journal.pone.0333029)
Supplement: S2 Table — (DOCX) [file pone.0333029.s002.docx]

**Motor performance as a predictor of blood levels of ammonia and inflammatory biomarkers in patients with liver cirrhosis**

Constanza San Martín Valenzuela^¶^, Juan José Gallego^¶^, Amparo Urios, Patricia Correa-Ghisays, Rafael Tabares-Seisdedos^*^, Carmina Montoliu^*^

**S2 Table. Blood ammonia and plasma levels of interleukins and chemokines tested in the sample of participants and a control group as a reference.**

| Plasma measurements | Control  (N=98) | cirrhosis  (n=67) | *P value* |
| --- | --- | --- | --- |
| IL-6 (pg/mL) | 0.99 ± 0.11 | 5.7 ± 0.4 | <0.0001 |
| IL-18 (pg/mL) | 139 ± 10 | 548 ± 43 | <0.0001 |
| il-13 (pg/mL) | 1.79 ± 0.16 | 6.8 ± 0.6 | <0.0001 |
| IL-21 (pg/mL) | 81.4 ± 18.2 | 801± 42 | <0.0001 |
| IL-22 (pg/mL) | 58.5 ± 2.8 | 89 ± 7 | <0.0001 |
| IL-23 (pg/mL) | 4.49 ± 1.13 | 19.8 ± 1.4 | <0.0001 |
| TNF-α (pg/mL) | 1.44 ± 0.06 | 2.9 ± 0.2 | <0.0001 |
| TGF-β (pg/mL) | 31240 ± 1404 | 6885 ± 431 | <0.0001 |
| CCL20 (pg/mL) | 11.15 ± 1.24 | 98 ± 9 | <0.0001 |
| CX3CL1 (pg/mL) | 530± 20 | 3492 ± 143 | <0.0001 |
| CXCL13 (pg/mL) | 60.42 ± 2.89 | 193 ± 9 | <0.0001 |
| CCL2 (pg/mL) | 5.1 ± 0.8 | 31 ± 2 | <0.0001 |
| AMMONIA (µM) | 11 ± 0.5 | 32 ± 4 | <0.0001 |

Blood test outcome values are expressed as mean ± standard error. The control data are from a previous publication (*Mangas-Losada et al. Sci. Rep. 2017; 7: 6683*). Comparisons between participants with cirrhosis and healthy controls were conducted using an unpaired t-test. Differences between cirrhotic participants and healthy volunteers were considered statistically significant when *p* was less than 0.05. Abbreviations: IL, interleukin; TNF, tumor necrosis factor; TGF, transforming growth factor; CCL; C-C motif chemokine ligand; CX3CL1, C-X3-C motif ligand 1; CXCL13, C-X-C motif ligand 13.
