# Supplementary material for: Motor performance as a predictor of blood levels of ammonia and inflammatory biomarkers in patients with liver cirrhosis
Source: PLoS One. 2025 Oct 8;20(10):e0333029. doi: 10.1371/journal.pone.0333029 (PMC12507304; doi:10.1371/journal.pone.0333029)
Supplement: S3 Table — (DOCX) [file pone.0333029.s003.docx]

**Motor performance as a predictor of blood levels of ammonia and inflammatory biomarkers in patients with liver cirrhosis**

Constanza San Martín Valenzuela^¶^, Juan José Gallego^¶^, Amparo Urios, Patricia Correa-Ghisays, Rafael Tabares-Seisdedos^*^, Carmina Montoliu^*^

**S3 Table.** **Differences between sample participants with and without diabetes on parameters from blood test**

| **Outcome from blood test** | ***p*-value** |
| --- | --- |
| AMONIO | .061 |
| IL6 | .249 |
| IL18 | .105 |
| IL13 | .079 |
| CCL20 | .793 |
| TNFα | .136 |
| IL22 | .148 |
| TGFβ | .378 |
| IL21 | .390 |
| IL23 | .139 |
| CX3CL1 | .554 |
| CXCL13 | .063 |
| CCL2 | .148 |

The table shows the p value of the statistical comparison performed through a Multivariate Analysis of Variance using the Diabetes factor as the independent variable between subjects. The number of participants with diabetes was *n*=26, while the number of participants without diabetes was *n*=41.
