# Supplementary material for: Motor performance as a predictor of blood levels of ammonia and inflammatory biomarkers in patients with liver cirrhosis
Source: PLoS One. 2025 Oct 8;20(10):e0333029. doi: 10.1371/journal.pone.0333029 (PMC12507304; doi:10.1371/journal.pone.0333029)
Supplement: S4 Table — (DOCX) [file pone.0333029.s004.docx]

**Motor performance as a predictor of blood levels of ammonia and inflammatory biomarkers in patients with liver cirrhosis**

Constanza San Martín Valenzuela^¶^, Juan José Gallego^¶^, Amparo Urios, Patricia Correa-Ghisays, Rafael Tabares-Seisdedos^*^, Carmina Montoliu^*^

**S4 Table. Differences between sample participants with and without diabetes on motor outcomes**

| **Gait assessment** | | | | | | | | | | | | |
| --- | --- | --- | --- | --- | --- | --- | --- | --- | --- | --- | --- | --- |
| **Ourcomes** | | | | ***p*-value** | | | **Outcomes** | | | ***p*-value** | | |
| Gait velocity (m/s) | | | | .142 | | | Propulsive force (N) | | | .949 | | |
| Stance time (s) | | | | .273 | | | Push-off force (N) | | | .352 | | |
| Braking force (N) | | | | .514 | | | Swing force (N) | | | .659 | | |
| **Balance assessment** | | | | | | | | | | | | |
| **Ourcomes** | | | | | | ***p*-value** | **Outcomes** | | | ***p*-value** | | |
| Romberg test with eyes open | Total CoP displacement | | | | | .295 | Romberg test with eyes closed | Total CoP displacement | | .349 | | |
|  | CoP displacement angle | | | | | .514 |  | CoP displacement angle | | .210 | | |
|  | ML CoP dispersion | | | | | .758 |  | ML CoP dispersion | | .927 | | |
|  | AP CoP dispersion | | | | | .801 |  | AP CoP dispersion | | .681 | | |
|  | CoP swept area | | | | | .509 |  | CoP swept area | | .443 | | |
|  | CoP velocity (m/s) | | | | | .346 |  | CoP velocity (m/s) | | .292 | | |
|  | ML CoP displacement | | | | | .973 |  | ML CoP displacement | | .876 | | |
|  | AP CoP displacement | | | | | .918 |  | AP CoP displacement | | .597 | | |
|  | ML CoP force | | | | | .316 |  | ML CoP force | | .519 | | |
|  | AP CoP force | | | | | .477 |  | AP CoP force | | .676 | | |
| Romberg test with eyes open and foam pad | Total CoP displacement | | | | | .790 | Romberg test with eyes closed and foam pad | Total CoP displacement | | .531 | | |
|  | CoP displacement angle | | | | | .976 |  | CoP displacement angle | | .235 | | |
|  | ML CoP dispersion | | | | | .728 |  | ML CoP dispersion | | .762 | | |
|  | AP CoP dispersion | | | | | .706 |  | AP CoP dispersion | | .839 | | |
|  | CoP swept area | | | | | .983 |  | CoP swept area | | .585 | | |
|  | CoP velocity (m/s) | | | | | .396 |  | CoP velocity (m/s) | | .853 | | |
|  | ML CoP displacement | | | | | .597 |  | ML CoP displacement | | .328 | | |
|  | AP CoP displacement | | | | | .232 |  | AP CoP displacement | | .223 | | |
|  | ML CoP force | | | | | .601 |  | ML CoP force | | .173 | | |
|  | AP CoP force | | | | | .717 |  | AP CoP force | | .157 | | |
| **Hand motor speed assessment** | | | | | | | | | | | | |
| **Outcome** | | | | | ***p*-value** | | **Outcome** | | | | ***p*-value** | |
| Unilateral speed left hand | | | | | .683 | | Bilateral speed left hand | | | | .699 | |
| Unilateral speed right hand | | | | | .693 | | Bilateral speed right hand | | | | .952 | |
| **Hand strength assessment** | | | | | | | | | | | | |
| **Outcome** | | ***p*-value** | **Outcome** | | | | | ***p*-value** | **Outcome** | | | ***p*-value** |
| Grip strength right | | .329 | Lateral pinch strength right | | | | | .531 | Tip pinch strength right | | | .731 |
| Grip strength left | | .709 | Lateral pinch strength left | | | | | .778 | Tip pinch strength left | | | .850 |
| Grip strength CV right | | .388 | Lateral pinch strength CV right | | | | | .307 | Tip pinch strength CV right | | | .666 |
| Grip strength CV left | | .538 | Lateral pinch strength CV left | | | | | .206 | Tip pinch strength CV left | | | .194 |
| Grip strength ID | | .242 | Lateral pinch strength ID | | | | | .321 | Tip pinch strength ID | | | .515 |

The table shows the p value of the statistical comparison performed through a Multivariate Analysis of Variance using the Diabetes factor as the independent variable between subjects. The number of participants with diabetes was *n*=26, while the number of participants without diabetes was *n*=41. CV, Coefficient of variation. ID, Index of Difference.
