# Supplementary material for: Motor performance as a predictor of blood levels of ammonia and inflammatory biomarkers in patients with liver cirrhosis
Source: PLoS One. 2025 Oct 8;20(10):e0333029. doi: 10.1371/journal.pone.0333029 (PMC12507304; doi:10.1371/journal.pone.0333029)
Supplement: S5 Table — (DOCX) [file pone.0333029.s005.docx]

**Motor performance as a predictor of blood levels of ammonia and inflammatory biomarkers in patients with liver cirrhosis**

Constanza San Martín Valenzuela^¶^, Juan José Gallego^¶^, Amparo Urios, Patricia Correa-Ghisays, Rafael Tabares-Seisdedos^*^, Carmina Montoliu^*^

**S5 Table. Outcome predictors from the multiple regression models that explain under 50% of the variance in the biomarkers analyzed**

| **Biomarker** | **Predictor** | **Unstandardized** | | **Stand. CI** | | **t** | ***p*** |
| --- | --- | --- | --- | --- | --- | --- | --- |
|  |  | **β** | **SE** |  |  |  |  |
| IL-13 | CoP swept area in REO | .02 | .00 | .663 | .01 ; .04 | 4.51 | .00 |
|  | CoP total Displacement in RFO | .51 | .10 | .707 | .73 ; .29 | 4.69 | .00 |
|  | AP CoP Dispersion in RFO | .51 | .15 | .486 | .19 ; .83 | 3.24 | .00 |
|  | ML CoP Dispersion in REC | .89 | .29 | .447 | 1.48 ; .29 | 2.99 | .00 |
|  | Braking force (CV) during gait | .16 | .07 | .217 | .00 ; .31 | 2.09 | .04 |
| IL-21 | Gait speed | -623.41 | 163.06 | -.39 | -949.59 ; -297.23 | -3.82 | .00 |
|  | AP CoP Force in RFC | 5.33 | 2.13 | .25 | 1.07 ; 9.59 | 2.50 | .01 |
|  | Lateral pinch strength ID | 5.25 | 2.01 | .26 | 1.21 ; 9.29 | 2.60 | .01 |
|  | Right lateral pinch strength (CV) | 31.16 | 12.76 | .25 | 5.63 ; 56.68 | 2.44 | .01 |
| CX3CL1 | Left-hand speed (unilateral) | -35.08 | 8.90 | -.43 | -52.896 ; -17.282 | -3.94 | .00 |
|  | CoP displacement angle in RFC | 4.99 | 1.29 | .41 | 2.408 ; 7.588 | 3.85 | .00 |
|  | AP CoP Dispersion in REC | 151.70 | 58.02 | .27 | 35.680 ; 267.728 | 2.61 | .01 |
| IL6 | Gait speed | -7.54 | 1.57 | -.50 | -10.68 ; -4.39 | -4.79 | .00 |
|  | CoP velocity in RFC | 31.13 | 12.42 | .26 | 6.30 ; 55.96 | 2.50 | .01 |
| CCL2 | CoP velocity in in RFC | 221.99 | 77.02 | .31 | 67.91 ; 376.07 | 2.88 | .00 |
|  | Left-hand speed (unilateral) | -.80 | .23 | -.57 | -1.27 ; -.32 | -3.39 | .00 |
|  | Left distal pinch strength (CV) | 1.26 | .47 | .29 | 2.21 ; .32 | 2.69 | .01 |
|  | Right-hand speed (unilateral) | -.40 | .18 | -.36 | -.02 ; -.77 | -2.14 | .03 |
| The standardized and unstandardized β coefficients, their standard error (SE), t values, significance, and confidence interval (CI) are reported for each predictor. ID, Index of Difference; CV, Coefficient of variation. Abbreviations from balance test: CoP, center of pressure; AP, anteroposterior direction; ML, mediolateral direction; GRF, ground reaction force; REO, Romberg test with eyes open; REC, Romberg test with eyes closed; RFO, Romberg with foam pad and eyes open; RFC, Romberg test with foam pad and eyes closed. | | | | | | | |

**S5 Table (continuation). Outcome predictors from the multiple regression models that explain under 50% of the variance in the biomarkers analyzed (continuation)**

| **Biomarker** | **Predictor** | **Unstandardized** | | **Stand. CI** | | **t** | ***p*** |
| --- | --- | --- | --- | --- | --- | --- | --- |
|  |  | **β** | **SE** |  |  |  |  |
| CXCL13 | Gait speed | -103.50 | 35.11 | -.33 | -173.71 ; -33.29 | -2.94 | .00 |
|  | AP CoP Force in RFC | 1.34 | .45 | .32 | .44 ; 2.24 | 2.98 | .00 |
|  | Gait speed CV | 8.32 | 4.09 | .22 | .13 ; 16.50 | 2.03 | .04 |
| IL22 | AP CoP Displacement in REO | 3.22 | .73 | .47 | 1.76 ; 4.68 | 4.41 | .00 |
|  | ML CoP Displacement in RFO | -1.54 | .65 | -.25 | -2.84 ; -.23 | -2.35 | .02 |
| CCL20 | CoP total Displacement in REO | 2.86 | .82 | .38 | 1.20 ; 4.51 | 3.46 | .00 |
|  | CoP velocity in RFC | 723.12 | 302.28 | .26 | 118.66 ; 1327.58 | 2.39 | .02 |
|  | Gait speed | -82.78 | 38.36 | -.23 | -159.49 ; -6.07 | -2.15 | .03 |
| IL18 | Gait speed | -704.36 | 199.76 | -.44 | -1103.68 ; -305.03 | -3.52 | .00 |
|  | ML CoP Dispersion in RFO | -28.54 | 13.54 | -.26 | -55.62 ; -1.47 | -2.10 | .03 |
| TGF-β | Hand Grip strength ID | 32.68 | 12.61 | .30 | 57.89 ; 7.46 | 2.59 | .01 |
|  | AP CoP Force in REO | -307.55 | 145.91 | -.24 | -599.24 ; -15.86 | -2.10 | .03 |
| The standardized and unstandardized β coefficients, their standard error (SE), t values, significance, and confidence interval (CI) are reported for each predictor. ID, Index of Difference; CV, Coefficient of variation. Abbreviations from balance test: CoP, center of pressure; AP, anteroposterior direction; ML, mediolateral direction; GRF, ground reaction force; REO, Romberg test with eyes open; REC, Romberg test with eyes closed; RFO, Romberg with foam pad and eyes open; RFC, Romberg test with foam pad and eyes closed. | | | | | | | |
